# Depression as a Risk Factor for Mortality in Individuals with Diabetes: A Meta-Analysis of Prospective Studies

**DOI:** 10.1371/journal.pone.0079809

**Published:** 2013-11-21

**Authors:** Mareike Hofmann, Birgit Köhler, Falk Leichsenring, Johannes Kruse

**Affiliations:** 1 Department of Psychosomatic Medicine and Psychotherapy, University of Giessen, Giessen, Germany; 2 Department of Psychosomatic Medicine and Psychotherapy, University of Marburg, Marburg, Germany; Population Health and Preventive Medicine, Malaysia

## Abstract

**Objective:**

To quantify the impact of depression measured by self-reports and depression measured by clinical interview on all-cause mortality in individuals with diabetes and to analyze the strength of both associations, the influence of covariates, and possible differences between studies assessing self-rated depressive symptoms and those using a clinical interview to measure depression as predictors of mortality.

**Research Design and Methods:**

PUBMED and PsycINFO were searched up to July 2013 for prospective studies assessing depression, diabetes and mortality. The pooled hazard ratios were calculated using random-effects models.

**Results:**

Sixteen studies met the inclusion criteria. After adjustment for demographic variables depression measured by self-reports was associated with an increased all-cause mortality risk (pooled HR = 2.56, 95% CI 1.89–3.47), and the mortality risk remained high after additional adjustment for diabetes complications (HR = 1.76, 95% CI 1.45–2.14,). Six studies reporting adjusted HRs for depression measured by clinical interviews supported the results of the other models (HR = 1.49, 95% CI 1.15–1.93).

**Conclusions:**

Both depression measured by self-report and depression measured by clinical interview have an unfavorable impact on mortality in individuals with diabetes. The results, however, are limited by the heterogeneity of the primary studies. It remains unclear whether self-reports or clinical interviews for depression are the more precise predictor.

## Introduction

Studies have confirmed that individuals with diabetes are more likely to show a depressive symptomatology than individuals without diabetes [1]–[3]. The mechanisms linking these two comorbid conditions with an increase of mortality are not clear yet and further research is needed [4].

Two recent meta-analyses support the hypothesis of a bi-directional relationship between diabetes and depression, indicating that depression itself is a risk factor for the development of diabetes [5], [6]. Depression was associated with a 60% increased risk of type 2 diabetes mellitus (pooled risk ratio [RR] 1.60, 95% confidence interval [CI] 1.37–1.88) whereas type 2 diabetes mellitus was associated with an only modest (pooled RR 1.15, 95% CI 1.02–1.30) risk of depression [6].

Comorbid depression increases the mortality risk among people with diabetes [7]–[9]. One study showed a hazard ratio (HR) for all-cause mortality of 1.88 (95% CI 1.55–2.27) in individuals with diabetes only, compared to 2.50 (95% CI 2.04–3.08) in individuals with coexisting diabetes and depression [9]. Interestingly, according to one study, lifetime depressive disorder, as measured with clinical diagnostic criteria, was a similar predictor for mortality (HR 4.59, 95% CI 2.12–9.93) than self-reported depressive symptoms (HR 4.94, 95% CI 3.30–7.38) [10]. The authors found a gradient response in the sense of a rising mortality risk associated with an increasing severity of depression in individuals with diabetes.

Despite the empirical evidence that depressive symptoms or depression in diabetes increase the risk of mortality, there are different pathways explaining the underlying mechanisms:

“Direct” effects of depression on physiological factors may lead to an increase in classic metabolic risk factors, which in turn may cause diabetes.

“Indirect” pathways refer to psychosocial and behavioural covariables, which correlate with depression and diabetes. Depression is associated with poor health behaviour, maladaptive coping style, social isolation, and chronic life stress [11]. Behavioural risk factors like smoking, a poor diet, and a low adherence to medical recommendations influence the relationship of depressive symptoms and diabetes [12]. Coexisting depression and diabetes are associated with changes in the neuroendocrine system, increased immunoinflammatory activation [13], an increased risk of diabetes complications [14], and poor adherence to diabetes management as well as poor glycemic control [15], [16].

Two recent meta-analyses showed similar estimates of risk of comorbid depression on mortality in diabetes. Van Dooren and colleagues [17] analysed that depression was associated with an increased risk of all-cause mortality (HR = 1.46, 95% CI 1.29–1.66), and cardiovascular mortality (HR = 1.39, 95% CI 1.11–1.73). Sixteen studies were included, five of them also reported on cardiovascular mortality. Heterogeneity across studies was high for all-cause mortality and relatively low for cardiovascular mortality.

Park et al. [18] included a total of 42.363 respondents from 10 studies in their analysis. Depression was significantly associated with the risk of mortality (pooled HR = 1.50, 95% CI 1.35–1.66), little evidence for heterogeneity was found across the studies. Both meta-analyses did not differentiate between assessments of depression or between adjustments for known covariates.

However, the question whether there is a difference between self-reported depressive symptoms or the clinical diagnoses of depression being associated with mortality in people with diabetes has not been subject of a meta-analysis. First, our objective was to examine whether depression measured via self-report or clinical interview increases the risk of mortality in people with diabetes. Second, we analysed the influence of covariates on the association of depression, diabetes, and mortality.

## Methods

### Search Strategy

The databases PUBMED and PsycINFO were searched for English and German language studies through July 30, 2013 We used the combination of 1) “depression” (title/abstract) or “depressive” (title/abstract) or “depressive disorder”; 2) “diabetes” (title/abstract) or “diabetes mellitus” (MeSH); 3) “mortality” (title/abstract) or “death” (title/abstract) or as medical subject headings; and 4) “longitudinal” (title/abstract) or “prospective” (title/abstract). The search was not restricted by publication language or by publication time. All findings were downloaded and stored in the reference program End Note 9. The search resulted in 130 references from PUBMED, 39 references from PsycINFO, and 7 references from other sources (30.07.2013). From the total of 176 references 147 references remained after deleting duplicates. The search was completed by cross-checking references listed in recently published meta-analyses [17], [18] and examining reference lists of published studies to obtain additional reports. Authors were contacted for additional information on mortality data.

### Inclusion Criteria

To be included, a study had to 1) be a prospective cohort study, 2) focus exclusively on patients diagnosed with either type 1 diabetes, type 2 diabetes, or any kind of diabetes or patients' self-reports on diabetes or report data of a subgroup with diabetic patients (at baseline), 3) assess either depressive symptoms or clinical depression by standardized self-report measures, questionnaires, or a standardized clinical interview, and 4) report results for depressed versus non depressed patients. Furthermore, 5) outcomes had to be a) death from any cause or b) death from cardiac cause, 6) the association of depressive symptoms or depressive disorders on mortality during follow-up had to be either provided or computable from the results section. For primary studies using the Cox regression model, the adjusted hazard ratios reported in the study were used.

Regarding multiple reports on the same dataset, only one report was included in the meta-analysis, based upon population size (largest sample size), the aim of the article (with mortality as the main end point), and length of follow-up.

### Data Extraction and Quality Assessment

After removing duplicates, 147 titles and abstracts were screened. 43 papers were included and assessed for eligibility by two independent authors (MH, BK), achieving an agreement of approximately 95%. Each eligible study was coded according to standardized criteria [19]: 1) citation of reference; 2) inclusion and exclusion criteria; 3) description of the patient sample (size of sample, age, sex, subgroups); 4) variables measured at baseline (depressive symptoms/clinical depression, diabetes); 5) type of outcome (all-cause mortality or cardiac mortality); 6) hazard ratios (HRs) with corresponding 95% CI; and 7) multivariable adjustment (e. g., age, sex, physical illness, smoking, hypertension).

**Figure 1:**
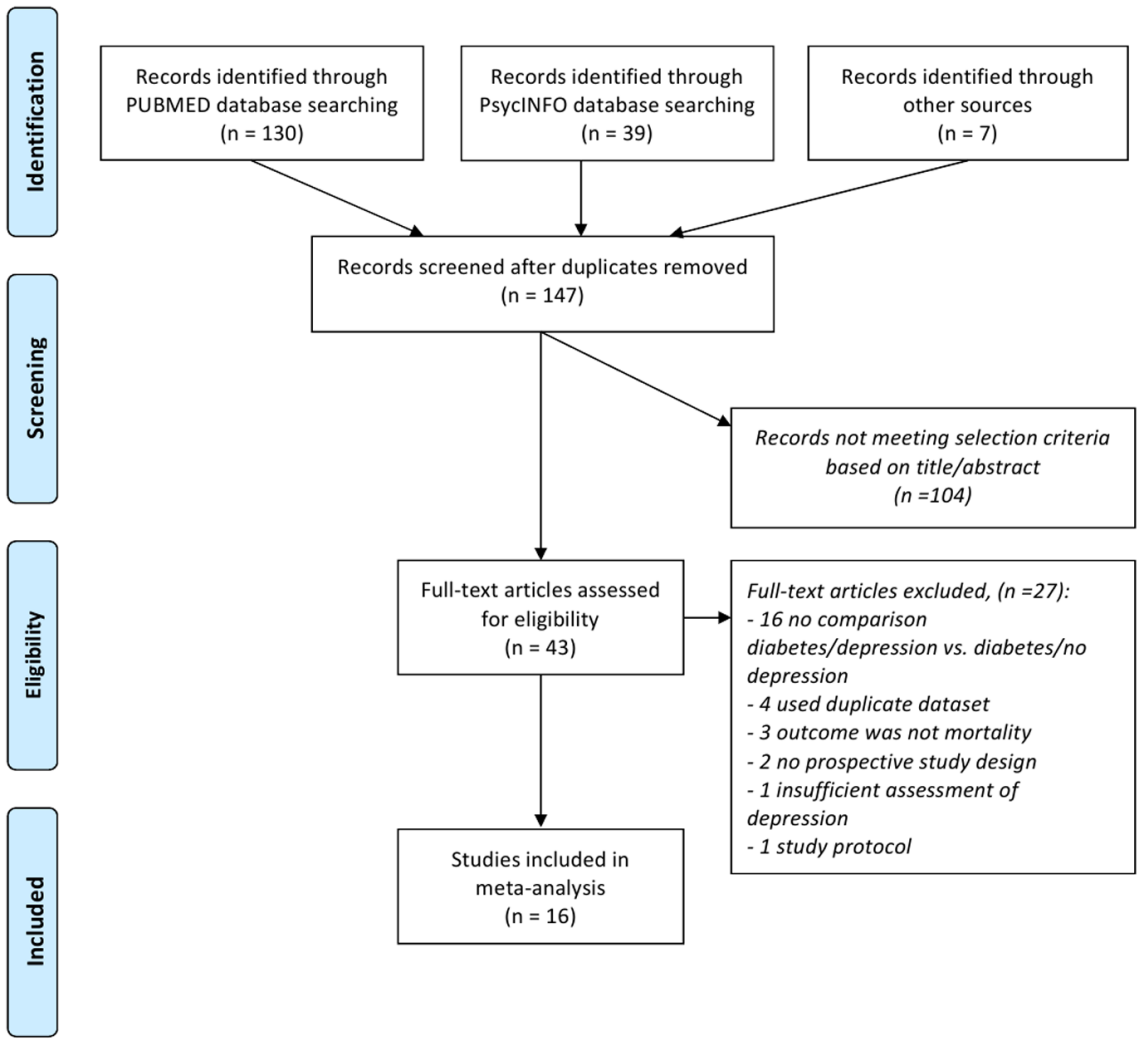
Flow of study selection.

In the end, 16 studies met the inclusion criteria (see Table 1). 27 papers had to be excluded (see Table S1 in File S1). Figure 1 illustrates the process of the study selection.

**Table 1:** Characteristics of studies in the meta-analysis in alphabetical order.

Authors, year, country	Number of participants (mean age, sex, type of diabetes)	Number of participants with depression (%)	Definition and assessment of depression, depressive symptoms vs. clinical depression	Selected estimate (95% CI)	Adjustment variables	Follow-up (years)
Ahola et al. [26] 2012, Finland	4,174 (39 years, 49% female, type 1)	313 (8%)	Purchase of antidepressant agents within 1 year prior to baseline (depressive symptoms)	a. 1.65 (1.12–2.42), 2.00 (1.34–3.00); b. 1.12 (0.71–1.77), 2.15 (1.34–3.45)	a. age, diabetes duration; b. age, diabetes duration, diastolic blood pressure, smoking, HbA_1c_, nephropathy	9
Black et al. [10] 2003, USA	636 (≥65 years, 59% female for total sample, type 2)	188 (30%)	Baseline-measurement: Center of Epidemiological Studies-Depression Scale (CES-D), cut-off ≥16 (depressive symptoms); assessment at the second wave (first follow-up interview): Composite International Diagnostic Interview (CIDI) (clinical depression)	depressive symptoms: 4.94 (3.30–7.38); clinical depression: 4.59 (2.12–9.93)	sex, age, education, acculturation, marital status	7
Bot et al. [25] 2012, The Netherlands	330 (65 years, 30% female, type not specified)	106 (32%)	Beck Depression Inventory score), cut-off ≥10 depressive symptoms)	a. 3.79 (2.71–5.29); b. 2.90 (2.07–4.07)	a. age, sex, smoking, hypertension; b. age, sex, smoking, hypertension, previous MI, Killip class, left ventricular ejection fraction	6.2
Bruce et al. [31] 2005, Australia	1,273 (64 years, 51% female, type 2)	401 (32%)	General Health Status Questionnaire (GHS) (depressive symptoms)	1.21 (0.95–1.55)	age, gender, diabetes duration, HbA_1c_, BP lowering therapy, BMI, smoking, exercise, indigenous Australian, CHD, CVD, Ln(ACR), retinopathy, neuropathy	7–8
Egede et al. [9] 2005, USA	715 (63 years, 62% female, type not specified)	262 (37%)	Center of Epidemiological Studies-Depression Scale (CES-D), cut-off ≥16 (depressive symptoms)	a. 3.27 (2.66–4.01); b. 2.50 (2.04–3.08)	a. age, sex, race/ethnicity, income ratio, education, marital status; b. smoking, physical activity, BMI, aspirin use, comorbid conditions (cancer, hypertension, heart disease, stroke)	8
Iversen et al. [23] 2009, Norway	1,494 (66 years, 50% female, type 1 and type 2)	258 (17%)	Hospital Anxiety and Depression Scale-D (HADS-D), cut-off ≥8, (depressive symptoms)	a. 1.37 (1.10–1.72); b. 1.35 (1.08–1.69)	a. age, male sex, education, smoking, waist circumference; b. microalbuminuria, HbA_1c_, insulin use	10
Katon et al. [33] 2008, USA	10,704 (76 years, 44% female, type not specified)	1,657 (15%)	ICD-9 depression codes from medicare claims data (clinical depression)	1.36 (1.16–1.59)	age, gender, race/ethnicity, Charlson score, prior CVA, CVD, or CVD procedure or amputation	2
Lin et al. [29] 2009, USA	4,184 (64 years, 49% female, type 2)	850 (20%)	Patient Health Questionnaire-9 (PHQ-9), minor vs. major depression (depressive symptoms)	a. 2.26 (1.79–2.85); b. 1.52 (1.19–1.95)	a. demographic characteristics; b. demographic, clinical characteristics, health habits and disease control measures	5
Pan et al. [32] 2011, USA	4,873 (67 years, 100% female, type 2)	1,000 (21%)	Mental Health Index (MHI-5), cut-off = 52 and/or physician-diagnosed depression and/or antidepressant medication (depressive symptoms)	1.90 (1.63–2.21) = RR	age, family history of diabetes and cancer, parental history of MI, marital status, ethnicity, BMI, physical activity, alcohol consumption, smoking, multivitamin use, estrogen hormone use, aspirin use, hypertension, hypercholesterolemia, heart disease, stroke, cancer	6
Pieper et al. [34] 2011, Germany	1,141 (66 years, 52% female, type 2)	165 (14%)	Depression Screening Questionnaire (DSQ), cut-off = 8 (depressive symptoms)	a. 2.49 (1.45–4.28); b. 2.14 (1.06–5.37)	a. age, sex; b. age, sex, waist circumference, school years, education, smoking, physical activity, malnutrition, triglycerides, HDL, hypertension, compliance	3.5
Richardson et al. [27] 2008, USA	14,500 (59 years, 0% female type 2)	806 (6%)	ICD-9 codes (clinical depression)	1.60 (1.3–1.8)	age, ethnicity, marital status, employment status, CHD, hypertension, stroke, cancer	10
Rosenthal et al. [21] 1998, USA	135 (70 years, 4% female, type not specified)	45 (33%)	Yesavage Depression Inventory; Geriatric Depression Scale (GDS), cut-off = 10 (depressive symptoms)	4.50 (1.47–13.82) OR converted to RR	-	3
Scherrer et al. [22] 2011, USA	53,632 (56 years, 12% female for total sample, type 2)	12,679 (24%)	ICD-9-CM codes (clinical depression)	1.06 (0.96, 1.17) OR converted to RR	-	7
Sullivan et al. [30] 2012, USA+ Canada	2,053 (62 years, 40% female, type 2)	624 (31%)	Patient Health Questionnaire-9 (PHQ-9), cut-off ≥10, probable major depression, probable minor depression, and continuous PHQ-9 score (depressive symptoms)	1.76 (1.12–2.78)	age, sex, race/ethnicity, primary/secondary CVD prevention, HbA_1c_, lipids, blood pressure, BMI, smoking, alcohol consumption, living alone, blood pressure, presence of microvascular complications, CHF, education, duration of diabetes, antidepressant medications, glucose, blood pressure and lipid medications, assignment to one of eight study intervention arms	4.7
Ting et al. [28] 2013, China	7,835 (57 years, 53% female, type 2)	153 (2%)	ICD-9 codes by psychiatrists and usage of psychotropic drug at enrolment to the Registry (clinical depression)	0.96 (0.55–1.66)	age, sex, smoking, duration of diabetes, BMI, systolic and diastolic blood pressure, HbA_1c_, lipids (LDL-cholesterol, HDL cholesterol, triglycerides)	7.4
Winkley et al. [24] 2012, United Kingdom	253 (62 years, 36% female, type 1 and type 2 [83%])	82 (32%)	Schedules for Clinical Assessment in Neuropsychiatry (SCAN) 2.1/DSM-IV (Diagnostic and Statistical Manual 4th edition) criteria for (clinical depression)	2.09 (1.34–3.25)	age, sex, marital status, socioeconomic status, smoking, mean HbA_1c_, diabetes complications, ulcer severity	5

Study quality, participants/outcome assessment, incomplete outcome, and selective outcome reporting was assessed using the Cochrane Collaboration's [20] tool.

### Statistical Analysis

Data were extracted independently by two reviewers (MH, BK). Differences were solved by discussion with a third co-author (JK) and ended in one final coding. HRs were used and computed by an estimate of the effect sizes and the standard error of the effect size. Effect size was computed by using the adjusted or non-adjusted HR.

Data management and data analysis were performed with Review Manager 5.1, provided by the Cochrane Collaboration (www.cochrane.org). When adequate, the meta-analysis will be performed using the fixed effects or the random effects model: in case of heterogeneity the random effects model will be used. If there is homogeneity (or heterogeneity under 50%) the fixed effects model will be used. Studies were weighted by the inverse variance weight. Summary statistics were reported as HR with a CI of 95%. Values greater than 1 indicate an unfavorable impact of clinical depression or depressive symptoms on mortality in patients with diabetes.

Heterogeneity between studies was assessed by examining forest plots of studies, by calculating a chi-square heterogeneity test, and through *I* ^2^ statistics. The chi-square value tests for statistically significant heterogeneity among trials; *p* values lower than .05 indicate heterogeneity; additionally higher *I* ^2^ values indicate greater variability among trials than would be expected by chance alone (range, 0–100%). The results were clustered for self-reported depressive symptoms and clinical diagnosis of depression (measured by clinical interview) as well as different covariates, i. e. for a) demographic variables and behavioural risk factors like smoking or activity as well as for b) additional diabetes-related risk factors. Funnel plots of all outcome measures can be found in the electronic supplementary material.

## Results

### Description of the Studies

Sixteen studies were included in the meta-analysis, representing 107944 individuals with diabetes and including 19589 (18.2%) with comorbid depression. The publications dated from 1998 to 2013 and were, with the exception of one in German, all published in English. The majority of the studies were conducted in the United States. The number of participants ranged from 135 [21] to 53,632 adults with diabetes [22]. Table 1 shows the main characteristics of the included studies as well as the selected estimates and adjustments.

### Type of Participants

Most of the studies (*n* = 9) included participants with type 2 diabetes, one study with type 1 diabetes only, two studies reported results of participants with type 1 and type 2 diabetes, and four studies did not specify. Two studies investigated patients with a diabetic foot ulcer [23], [24], one study included patients with an additional coronary heart disease [9], and one study examined the mortality risk after myocardial infarction [25]. The majority of the participants were male (mean 57%). The mean age at baseline ranged from 56 [22] to 76 years [8], with exception of the only study on patients with type 1 diabetes only with a mean age of 39 years [26].

### Assessment of Depression

Five studies solely assessed clinical depression, ten solely depressive symptoms, and one assessed both, clinical depression and depressive symptoms [10].

Three studies used ICD-9 criteria to assess clinical depression, one as part of further measurements using codes from medicare claims data [8], one with the help of a previously validated enhanced algorithm to identify depression [27], and one by psychiatrists diagnosis [28]. One study measured clinical depression with the Schedules for Clinical Assessment in Neuropsychiatry 2.1 (SCAN 2.1) [24].

The Centre of Epidemiological Studies-Depression Scale (CES-D) was applied in two studies to measure depressive symptoms [9], [10], the Patient Health Questionnaire (PHQ-9) was used in two studies [29], [30], and another study used the General Health Status Questionnaire (GHS) [31] with symptoms presented on a visual analogue scale. One study assessed depression by way of self-report (Mental Health Index/MHI-5) and/or physician-diagnosed depression and/or antidepressant medication [32]. This study was coded as measuring depressive symptoms (for more details see Table 1).

### Study Outcome

The outcome of the studies was either all-cause mortality, reported in all 16 studies, or cardiovascular mortality, reported in five papers [9], [25], [29], [31], [32]. One study distinguished between all-cause, cardiac, cancer and noncardiac/noncancer mortality [29]. We treated the results of Ahola et al. [26] as separate studies (male and female subgroups), instead of calculating pooled HRs. The length of follow-up varied from 2 [8] to 10 years [23], [27] with a mean follow-up of 6 years. Characteristics adjusted for varied among the studies.

### Effect of Self-Reported Depressive Symptoms

**Figure 2:**
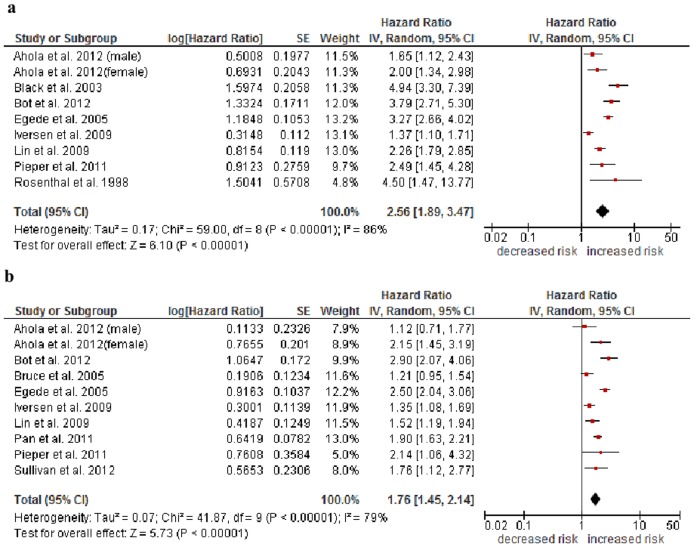
Forest plots of studies included in meta-analysis of all-cause mortality associated with depressive symptoms in individuals with diabetes.

After adjustment for demographic variables, individuals with diabetes and comorbid depressive symptoms have an increased mortality risk compared to those without depressive symptoms (pooled HR 2.56, 95% CI 1.89–3.47, *p*<.00001). This estimate is based on heterogeneous results (*I^2^* = 86%; Fig. 2a). After additional adjustment for clinical variables (e. g. BMI, hypertension, cancer, stroke) as well as diabetes-related variables (e. g. diabetes duration, HbA_1c_), the impact of depressive symptoms on mortality remained with a HR of 1.76 (95% CI 1.45–2.14, *p*<.00001). Again, there was substantial heterogeneity (*I^2^* = 79%, Fig. 2b).

### Effect of Clinical Diagnosis of Depression

**Figure 3:**
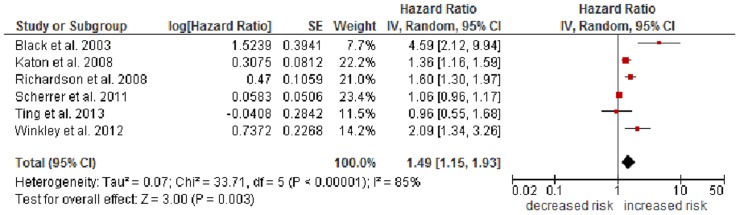
Forest plot of studies included in meta-analysis of all-cause mortality associated with clinical depression in individuals with diabetes.

The effect of clinical depression was assessed with adjusted HRs and an odds ratio (OR) converted to RR [22], which then can be combined with HRs in a meta-analysis as forms of relative risks. Figure 3 shows a significant effect of clinical depression on mortality with an HR of 1.49 (95% CI 1.15–1.93, *p*.00001). This effect is based on heterogeneous results (*I^2^* = 85%).

### Sensitivity Analysis

We conducted tests to assess the robustness of the findings above. First, fixed and random effects meta-analyses were undertaken for all three modalities. In two cases the random effects estimate was more beneficial (effect of depressive symptoms a. fixed model pooled HR = 2.40, 95% CI = 2.17–2.66 vs. random model pooled HR = 2.56, 95% CI = 1.89–3.47; effect of clinical depression fixed model pooled HR = 1.22, 95% CI = 1.13–1.32 vs. random model pooled HR = 1.49, 95% CI = 1.15–1.93). Further analysing possible small-study effects, we excluded poorer quality studies as they had only low sample sizes and large SDs. This did not change the effect size, either. In one case there was no substantial difference (fixed model pooled HR = 1.78, 95% CI = 1.64–1.93 vs. random model pooled HR = 1.76, 95% CI = 1.45–2.14). Second, the leave-one-out analyses found that no single report unduly influenced the pooled HR estimates of the association between depressive symptoms resp. clinical depression and mortality among those with diabetes. Funnel plots of the three analyses (see Figures S1a and S1b in File S1) suggest evidence of publication bias.

## Discussion

The results of this meta-analysis indicate that depression has a modest adverse effect on mortality in individuals suffering from diabetes. The negative effect of depression on diabetes can not only be found in more severe clinical cases of depression but also in individuals with mild depressive symptoms or subclinical depression. The adjustment of socio-demographic characteristics as well as clinical risk factors and diabetes complications results in a substantial higher relative mortality risk for depressed subjects with diabetes. In any case, the mortality rate is higher for persons with diabetes who report depressive symptoms than for those without depressive symptoms.

Our study extends the results of van Dooren et al. [17] and Park et al. [18] who showed that the coexistence of diabetes and depression is associated with significant mortality. The addition of latest data and the differentiation between self-reported depressive symptoms and the clinical diagnosis of depression increase the robustness of found effects and support us in demonstrating the impact of depression on mortality in individuals with diabetes more confidently.

There are sufficient data on the longitudinal impact of depressive symptoms on mortality. Results on the long-term effect of clinical depression, however, are limited. The study of Richardson et al. [27], who assessed male veterans over a 10-year period, provides initial hints, but further research is necessary to obtain more robust effects on the impact of depression on mortality in subjects with diabetes. The majority of the included study participants had type 2 diabetes, analyzing the effects of type 1 diabetes and depression on mortality would be also interesting. Furthermore, most of the included studies solely assessed all-cause mortality as end point; here a more distinct differentiation would be desirable to gain more precise information. It would also be helpful, if future research adjusted the analysis for risk factor information not only in the initial phase, but also during the follow-up.

In our meta-analysis, we differentiated between studies assessing depressive symptoms with self-report measures and those assessing a clinical diagnosis. The advantages and disadvantages of the respective measurement modality are known. Self-reports enable large screenings and may be more sensitive in detecting subthreshold disorders but cannot replace clinical diagnoses as derived from standardized diagnostic interviews. We did not detect a clear predictive difference concerning the use of self-report measurements and clinical interviews. Concerning the included studies of depressive symptoms it is important to keep in mind that using artificially dichotomized scores of continuous measures of depressive symptoms may have affected the results and therefore has to be regarded as a limitation of the available data. Furthermore we only found a small number of studies assessing depression via clinical interview. As these studies use different clinical interviews “clinical depression” does not mean the same thing from one study to the next. This may have contributed to the heterogeneity of the results, it can be considered as a further limitation of the available data.

The results presented in this study should be interpreted with caution when heterogeneity was observed. Obvious heterogeneity was present in studies adjusting for socio-demographic risk factors and analysing the impact of depressive symptoms. Contributing factors may be variable assessment of depression and diabetes, sample size, publication bias etc.

The strength of our meta-analysis is the inclusion of the most updated literature on the relationship between diabetes, depression and mortality, the use of explicit inclusion criteria and a strict procedure for data extraction. Additionally, we have integrated results of adjusted HRs which took into account baseline differences in relevant prognostic variables. These HRs more precisely represent the pure effect of depression and diabetes on mortality.

In conclusion, there appears to be an unfavorable effect of depressive symptoms as well as clinical depression on all-cause mortality in individuals with diabetes. More differentiated longitudinal studies would be helpful for further investigations.

## Supporting Information

Checklist S1 PRISMA Checklist.
(DOC)

File S1 **Table S1**, Description of excluded studies (n = 27).**Figure S1a**, Funnel plots of trials studying depressive symptoms as a risk factor for mortality: (**a**) adjusted (demographic) risk estimates using HR and (**b**) adjusted (demo+clinical characteristics+diabetic complications) risk estimates using HR. **Figure S1b**, Funnel plot of clinical depression as a risk factor for mortality.
(DOCX)
