# Supplementary material for: Depression as a Risk Factor for Mortality in Individuals with Diabetes: A Meta-Analysis of Prospective Studies
Source: PLoS One. 2013 Nov 21;8(11):e79809. doi: 10.1371/journal.pone.0079809 (PMC3836777; doi:10.1371/journal.pone.0079809)
Supplement: File S1 — Table S1, Description of excluded studies (n = 27).Figure S1a, Funnel plots of trials studying depressive symptoms as a risk factor for mortality: (a) adjusted (demographic) risk estimates using HR and (b) adjusted (demo+clinical characteristics+diabetic complications) risk estimates using HR. Figure S1b, Funnel plot of clinical depression as a risk factor for mortality. (DOCX) [file pone.0079809.s002.docx]

**Combined Supporting Information File S1**

**Table S1.** Description of excluded studies (n=27).

| **Citation** | **Reason for exclusion** |
| --- | --- |
| 1. Ariyo AA, Haan M, Tangen CM, Rutledge JC, Cushman M, et al. (2000) Depressive symptoms and risks of coronary heart disease and mortality in elderly Americans. Cardiovascular Health Study Collaborative Research Group. Circulation 102: 1773-1779. | No comparison of people with diabetes and depression vs. people with diabetes and without depression. |
| 2. Atlantis E, Grayson DA, Browning C, Sims J, Kendig H (2011) Cardiovascular disease and death associated with depression and antidepressants in the Melbourne Longitudinal Studies on Healthy Ageing (MELSHA). Int J Geriatr Psychiatry 26: 341-350. | No comparison of people with diabetes and depression vs. people with diabetes and without depression. |
| 3. Bai YM, Su TP, Chen MH, Chen TJ, Chang WH (2013) Risk of developing diabetes mellitus and hyperlipidemia among patients with bipolar disorder, major depressive disorder, and schizophrenia: A 10-year nationwide population-based prospective cohort study. J Affect Disord. | Outcome was risk of initiation of anti-diabetic or anti-hyperlipidemia medications not mortality. |
| 4. Baxter AJ, Charlson FJ, Somerville AJ, Whiteford HA (2011) Mental disorders as risk factors: assessing the evidence for the Global Burden of Disease Study. BMC Med 9: 134. | Article did not examine diabetes and depression comorbidity longitudinal. |
| 5. Bullard KM (2008) Biological and cultural influences in the relationship between depressive symptoms, type 2 diabetes risk, and all-cause mortality in older Mexican Americans. Dissertation Abstracts International: Section B: The Sciences and Engineering 68: 5167. | No comparison of people with diabetes and depression vs. people with diabetes and without depression. |
| 6. de Burgos-Lunar C, Gomez-Campelo P, Cardenas-Valladolid J, Fuentes-Rodriguez CY, Granados-Menendez MI, et al. (2012) Effect of depression on mortality and cardiovascular morbidity in type 2 diabetes mellitus after 3 years follow up. The DIADEMA study protocol. BMC Psychiatry 12: 95. | Study protocol |
| 7. Everson SA, Roberts RE, Goldberg DE, Kaplan GA (1998) Depressive symptoms and increased risk of stroke mortality over a 29-year period. Arch Intern Med 158:1133-1138 | The authors analyze the association between depressive symptoms and stroke mortality. No data about people with diabetes and depression.vs. people with diabetes without depression. |
| 8. Gallo JJ, Bogner HR, Morales KH, Post EP, Ten Have T, et al. (2005) Depression, cardiovascular disease, diabetes, and two-year mortality among older, primary-care patients. Am J Geriatr Psychiatry 13: 748-755. | The study does not analyze the interaction between people with diabetes and depression and nondepressed people with diabetes and mortality. |
| 9. Kamphuis MH, Geerlings MI, Giampaoli S, Nissinen A, Grobbee DE, et al. (2009) The association of depression with cardiovascular mortality is partly explained by health status. The FINE Study. J Affect Disord 114: 184-192. | This article analyzes to what extent subjective health status explained the association between depressive symptoms and cardiovascular mortality. No data about people with diabetes and depression vs. people with diabetes without depression. |
| 10. Katon W, Russo J, Lin EH, Heckbert SR, Ciechanowski P, et al. (2009) Depression and diabetes: factors associated with major depression at five-year follow-up. Psychosomatics 50: 570-579. | The authors assess the association between the risk of macrovasvular and microvascular complications and meeting criteria for major depression at 5-year follow-up. No comparison with nondepressed patients with diabetes is possible. Data on the impact of depression on mortality is also missing. |
| 11. Kellerman QD, Christensen AJ, Baldwin AS, Lawton WJ (2010) Association between depressive symptoms and mortality risk in chronic kidney disease. Health Psychol 29:594-600 | No data about people with diabetes and depression vs. people with diabetes without depression. |
| 12. Koopmans B, Pouwer F, de Bie RA, van Rooij ES, Leusink GL, et al. (2009) Depressive symptoms are associated with physical inactivity in patients with type 2 diabetes. The DIAZOB Primary Care Diabetes study. Fam Pract 26: 171-173. | No prospective study. |
| 13. Kuo Y-F, Raji MA, Peek MK, Goodwin JS (2004) Health-related social disengagement in elderly diabetic patients: association with subsequent disability and survival. Diabetes Care 27:1630-1637 | The authors do not assess depression by standardized measures or a clinical interview. |
| 14. Krause KJ, Bloom T (2012) Chronic condition mortality in the Medicare population. J Insur Med 43: 145-153. | No data about people with diabetes and depression vs. people with diabetes without depression. |
| 15. Lin EH, Rutter CM, Katon W, Heckbert SR, Ciechanowski P, et al. (2010) Depression and advanced complications of diabetes: a prospective cohort study. Diabetes Care 33: 264-269. | Same data set as in Lin et al. 2009 [29] which was included in the meta-analysis |
| 16. Martin S, Schramm W, Schneider B, Neeser K, Weber C, et al. (2007) Epidemiology of complications and total treatment costs from diagnosis of Type 2 diabetes in Germany (ROSSO 4). Exp Clin Endocrinol Diabetes 115: 495-501. | No data about people with diabetes and depression vs. people with diabetes without depression. |
| 17. Pina-Escudero SD, Navarrete-Reyes AP, Avila-Funes JA (2011) Depressive symptoms increase the risk of mortality in older Mexican community-dwelling adults. J Am Geriatr Soc 59: 2171-2172. | No data about people with diabetes and depression vs. people with diabetes without depression. |
| 18. Prisciandaro JJ, Gebregziabher M, Grubaugh AL, Gilbert GE, Echols C, Egede LE (2011) Impact of psychiatric comorbidity on mortality in veterans with type 2 diabetes. Diabetes Technology & Therapeutics 13:73-78 | The authors assess the impact of psychiatric comorbidity on mortality in veterans with type 2 diabetes. No data about the interaction between patients with diabetes and depression and nondepressed patients with diabetes and mortality. |
| 19. Reynolds SL, Haley WE, Kozlenko N (2008) The impact of depressive symptoms and chronic diseases on active life expectancy in older Americans. Am J of Geriatr Psychiatry 16:425-432 | Outcome parameter is active life expectancy which combines total life expectancy – the average number of years a person of a specific age can expect to live- and disabled life expectancy – the average number of those remaining years a person can expect to live with disability. Data on the impact of depression on mortality is missing. |
| 20. Rodriguez-Saldana J, Morley JE, Reynoso MT, Medina CA, Salazar P, Cruz E, et al. (2002) Diabetes mellitus in a subgroup of older Mexicans: prevalence, association with cardiovascular risk factors, functional and cognitive impairment, and mortality. J Am Geriatr Soc 50:111-116 | No data about people with diabetes and depression vs. people with diabetes without depression are analyzed separately. |
| 21. Takeshita J, Masaki K, Ahmed I, Foley DJ, Li YQ, Chen R, et al. (2002) Are depressive symptoms a risk factor for mortality in elderly Japanes American men?: the Honolulu-Asia Aging Study. Am J Psychiatry 159:1127-1132 | The authors assess the impact of depression on mortality in physically ill subjects. At baseline they analyze different groups of physically ill subjects seperately, e. g. persons with diabetes. At follow-up the authors only describe differences between a physically ill group and a physically healthy group. So there is no data about the interaction between subjects with diabetes and depression and nondepressed supjects with diabetes and mortality. |
| 22. Teng PR, Yeh CJ, Lee MC, Lin HS, Lai TJ (2013) Depressive symptoms as an independent risk factor for mortality in elderly persons: results of a national longitudinal study. Aging Ment Health 17: 470-478. | No data about the interaction between subjects with diabetes and depression and nondepressed people with diabetes and mortality. |
| 23. Whooley MA, Browner WS (1998) Association between depressive symptoms and mortality in older women. Study of Osteoporotic Fractures Research Group. Arch Intern Med 158:2129-2135 | No data about the interaction between subjects with diabetes and depression and nondepressed people with diabetes and mortality. |
| 24. Winkley K, Stahl D, Chalder T, Edmonds ME, Ismail K (2009) Quality of life in people with their first diabetic foot ulcer: a prospective cohort study. J Am Podiatr Med Assoc 99:406-414 | At follow-up the authors do not describe differences between persons with diabetes vs. persons without diabetes. So there is no data about the interaction between subjects with diabetes and depression and nondepressed subjects with diabetes and mortality. |
| 25. Winkley K, Stahl D, Chalder T, Edmonds ME, Ismail K (2007) Risk factors associated with adverse outcomes in a population-based prospective cohort study of people with their first diabetic foot ulcer. J Diabetes Complications 21:341-349 | Same data set as in Winkley et al. 2012 [24] which was included in the meta-analysis. |
| 26. Young BA, Von Korff M, Heckbert SR, Ludman EJ, Rutter C, et al. (2010) Association of major depression and mortality in Stage 5 diabetic chronic kidney disease. Gen Hosp Psychiatry 32: 119-124. | Special population with chronic kidney disease out of same data set (Pathway Study) as in Lin et al. 2009 [29] which was included in the meta-analysis. |
| 27. Zhang X, Norris SL, Gregg EW, Cheng YJ, Beckles G, Kahn HS (2005) Depressive symptoms and mortality among persons with and without diabetes. Am J Epidemiol 161:652-660 | This article describes the same results as in study by Egede et al. 2005 [9] which was included in the meta-analysis. |

**a**

**
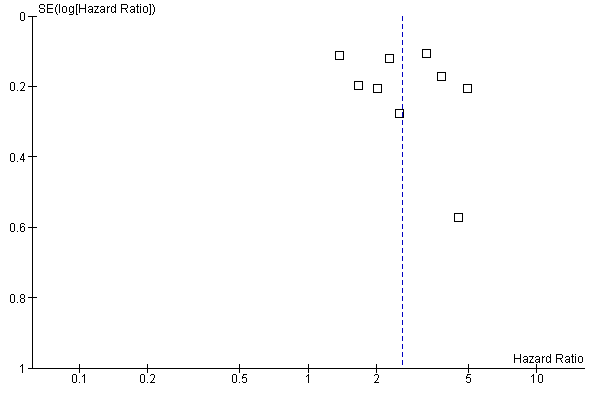
**

**b**

**
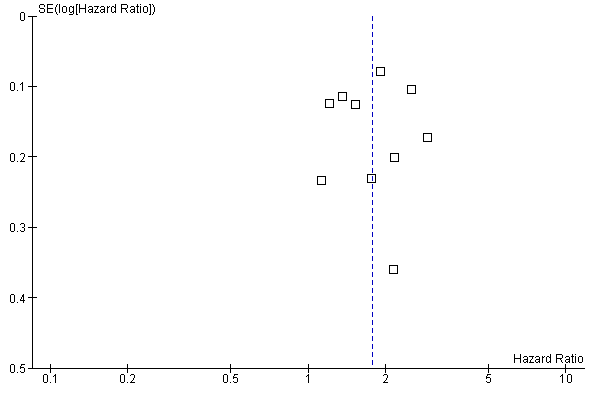
**

**Figure S1a.** Funnel plots of trials studying depressive symptoms as a risk factor for mortality: (**a**) adjusted (demographic) risk estimates using HR and (**b**) adjusted (demo + clinical characteristics + diabetic complications) risk estimates using HR.

**a**

**
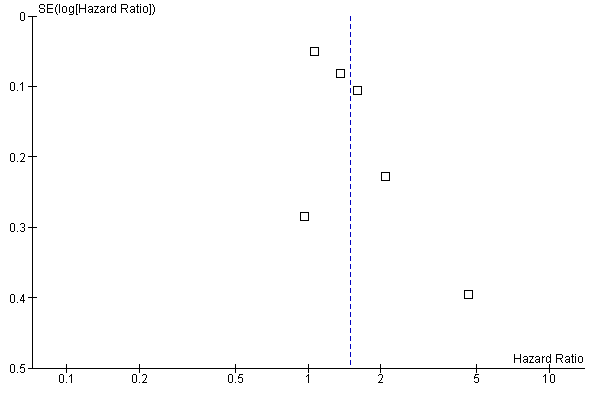
**

**Figure S1b.** Funnel plot of clinical depression as a risk factor for mortality.
